# Activation of Aurora A kinase increases YAP stability via blockage of autophagy

**DOI:** 10.1038/s41419-019-1664-4

**Published:** 2019-06-03

**Authors:** Peng Wang, Ying Gong, Tao Guo, Man Li, Lei Fang, Shengchen Yin, Muhammad Kamran, Yang Liu, Jie Xu, Lingzhi Xu, Fei Peng, Xiaoyuan Xue, Mengying Yang, Mie-Chie Hung, Eric W.-F. Lam, Chundong Gu, Chunli Wang, Qimin Zhan, Quentin Liu

**Affiliations:** 1 Institute of Cancer Stem Cell, Dalian Medical University, Dalian, China; 2 The First Affiliated Hospital, Dalian Medical University, Dalian, China; 3 Laboratory of Molecular Oncology, Peking University Cancer Hospital & Institute, Beijing, China; 4 The Second Affiliated Hospital, Dalian Medical University, Dalian, China; 5 Department of Molecular and Cellular Oncology, The University of Texas MD Anderson cancer center, Houston, TX 77030 USA; 6 Department of Surgery and Cancer, Imperial College London, Imperial Centre for Translational and Experimental Medicine (ICTEM), Du Cane Road, London, W12 0NN UK

**Keywords:** Non-small-cell lung cancer, Autophagy, HIPPO signalling

## Abstract

Transcription cofactor Yes-associated protein (YAP) plays an important role in cancer progression. Here, we found that Aurora A kinase expression was positively correlated with YAP in lung cancer. Aurora A depletion suppresses lung cancer cell colony formation, which could be reversed by YAP ectopic overexpression. In addition, activation of Aurora A increases YAP protein abundance through maintaining its protein stability. Consistently, the transcriptional activity of YAP is increased upon Aurora A activation. We further showed that shAURKA suppressed YAP expression in the absence of Lats1/2, indicating that Aurora A regulates YAP independently of Hippo pathway. Instead, Aurora A induced blockage of autophagy to up-regulate YAP expression. Collectively, our findings provide insights into regulatory mechanisms of YAP expression in lung cancer development.

## Introduction

The Hippo pathway functions as a key regulator of cell proliferation and organ size^1^. As the core component of Hippo pathway, the Yes-associated protein (YAP) shuttles between the cytoplasm and nucleus. In the nucleus, YAP interacts with DNA-binding transcription factors TEAD to promote the expression of several growth-related genes, including *CTGF* and *CYR61*^2,3^. The cellular location of YAP is determined by its phosphorylation status which is mainly regulated by upstream kinases in Hippo pathway^4–7^. In mammals, MST1/2 (mammalianSte20-like serine/threonine kinases1/2) and LATS1/2 (large tumor suppressor 1/2) are the central upstream kinases, inhibiting YAP transcriptional activity by phosphorylating YAP protein^8^. The phosphorylation of YAP at Serine 127 promotes 14-3-3 protein binding and restricts YAP to the cytoplasm, where its transcriptional function is shut down^9,10^. Another important phosphorylation site is Serine 397, which primes YAP for subsequent phosphorylation by casein kinase 1 (CK1), leading to its poly-ubiquitination and degradation^11–13^. Dysregulation of Hippo pathway exerts a significant impact on the development of a number of cancers, including lung cancer^14^, colorectal cancer^15^, ovarian cancer^16^ and liver cancer^17^. Furthermore, elevated expression and/or nuclear accumulation of YAP and TAZ are necessary to drive tumor initiation, progression, stemness, metastasis and immune evasion, including lung cancer, where YAP/TAZ role was extensively investigated^18^, pointing to the important role of YAP as a potent oncogene and therapeutic target in cancer^19–22^.

Recently, YAP has been reported to associate with Aurora A kinase by a pull-down assay and mass spectrometry analysis of YAP interacting proteins in the triple-negative MDA-MB-231 breast cancer cells^23^. Aurora A belongs to the Aurora family of serine/threonine kinases. The activation of Aurora A in late G2 phase of the cell cycle is essential for recruitment of the cyclin B1-Cdk1 complex to centrosomes, which is required for mitotic entry^24,25^. Aurora A overexpression has been found in a broad range of human malignancies, such as breast cancer^26^, lung cancer^27^, hepatocellular carcinoma^28^, gliomas^29^, gastric tumor^30^ and esophageal carcinoma^31^. Aurora A overexpression results in activation of PI3K/AKT, β-catenin and c-Myc, which promotes cancer cell proliferation, metastasis and self-renewal of cancer stem cells^32–35^. In addition, Aurora A can, through activating the mTOR pathway in breast cancer, block the autophagy, which has been proved to be essential for maintaining and promoting cellular homeostasis^36–38^. As a result, Aurora A is considered as a promising molecular target for cancer therapy^39–42^.

Lung cancer is one of the most prevalently diagnosed cancer types with high mortality rates^43^. Non-small cell lung cancers (NSCLCs) comprises approximately 85% of all lung cancers and have a poor survival rate, among which over 85% have an overall survival rate of less than 5-year^44^. Traditionally, the standard first-line treatment for advanced NSCLC is platinum-based chemotherapy regimens^45^. However, the therapeutic effects and long-term benefits of patients are limited, highlighting a substantial unmet clinical need. Further studies are required for uncovering the underlying mechanisms of lung cancer tumorigenesis and development.

Here we demonstrated that Aurora A functions as a positive upstream regulator of YAP to promote the proliferation and migration of lung cancer cells. Aurora A enhances YAP protein stability independently of the Hippo pathway and proteasome pathway. Activation of Aurora A kinase increases YAP stability via blockage of autophagy. These findings provide a new therapeutic avenue for lung cancer with Aurora A and YAP overexpression.

## Results

### Aurora A expression positively correlates with YAP protein level in lung cancer

**Fig. 1:**
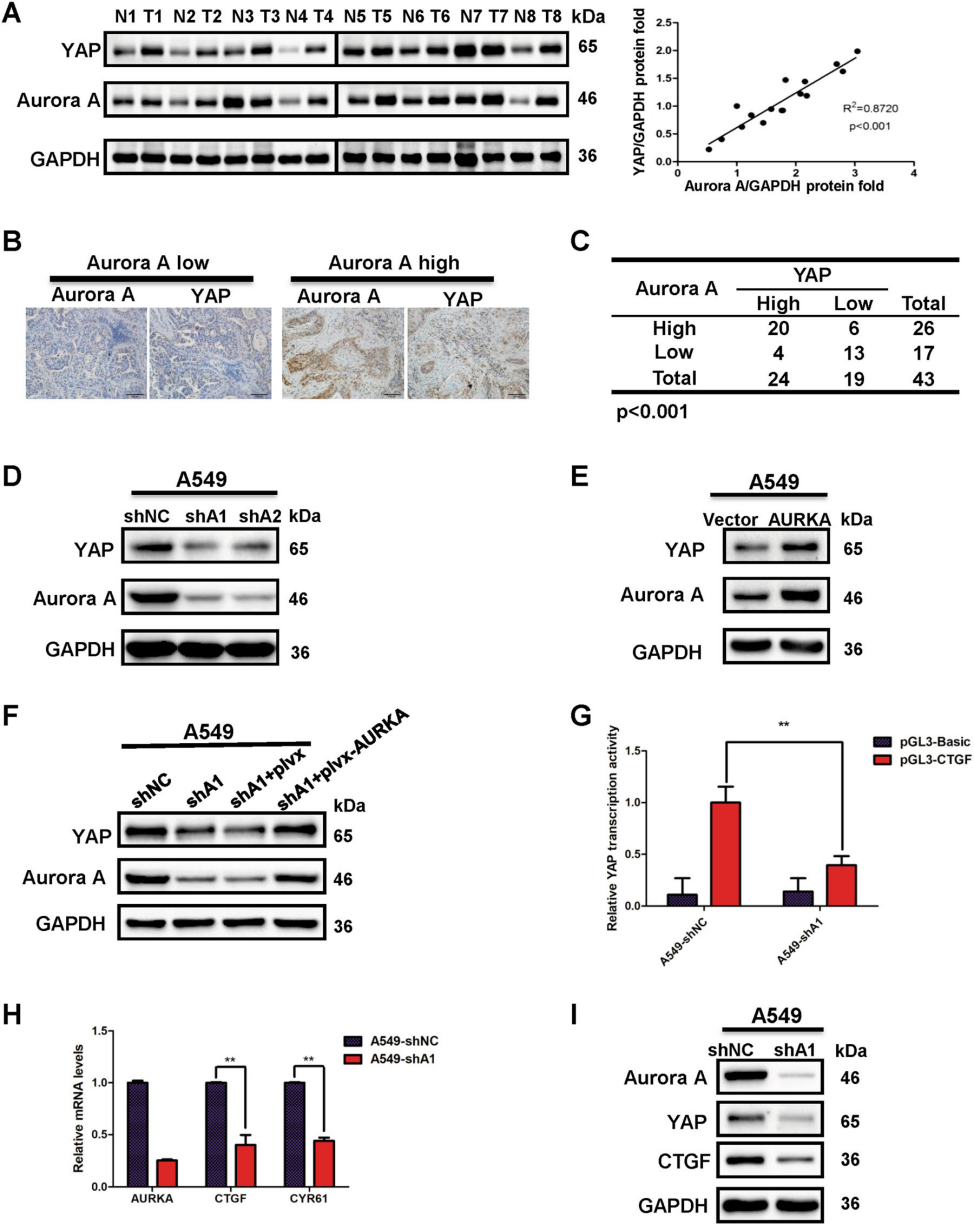
Aurora A regulates the protein expression and transcription activity of YAP. **a** Western blot analysis of the protein levels of Aurora A and YAP in eight pairs of clinical lung cancer (T) and adjacent normal tissues (N). Statistical analysis of Aurora A and YAP expression levels normalized to relative glyceraldehyde 3-phosphate dehydrogenase (GAPDH) (*R*^2^ = 0.8720, ***P < 0.001). **b** Human lung cancer tissues were analyzed by immunohistochemical staining with Aurora A and YAP antibody. Representative images were presented. Scale bars, 100 μm. **c** The correlation between Aurora A and YAP expression in lung cancer tissues from 43 patients. ***P < 0.001. **d** Western blot analysis of YAP protein expression in A549 cells with Aurora A knockdown (shA1 or shA2). **e** Western blot analysis of YAP protein expression in A549 cells with Aurora A overexpression (AURKA). **f** Aurora A depleted A549 cells were transfected with plvx or plvx-AURKA plasmids. Western blot analysis of YAP protein expression. **g** Luciferase reporter assay to evaluate the activity of YAP from A549 cells with Aurora A knockdown(shA1). Error bars represented mean ± S.D. (*n* = 3, **P < 0.01). **h** RT-qPCR analysis of the mRNA levels of YAP target genes *(CTGF, CYR61*) in shAURKA (shA1) and shNC A549 cells. Error bars represented mean ± S.D. (*n* = 3, **P < 0.01). **i** Western blot analysis of the protein levels of YAP and its target gene CTGF in shAURKA (shA1) and shNC A549 cells

To uncover the correlation between Aurora A and YAP, we conducted western blot analysis of YAP and Aurora A expression in eight pairs of clinical lung cancer and adjacent normal tissues (Fig. 1a, left panel). Compared with adjacent normal tissues, Aurora A and YAP protein levels were up-regulated at varying degrees. The expression of YAP was relatively higher in lung cancer tissues which showed much more abundant Aurora A expression. And the statistical analysis revealed a positive correlation between Aurora A and YAP protein expression (R^2^ = 0.8720, *p* < 0.001) (Fig. 1a, right panel), indicating that YAP overexpression is associated with high Aurora A levels in clinical lung cancer tissues. Furthermore, we assessed both Aurora A and YAP expression levels in 43 clinical lung cancer tissue specimens using immunohistochemistry (Fig. 1b, c and Supplementary Table 1). Of 43 patients, 33(76.7%) tissue specimens showed simultaneously high-expression or low-expression of Aurora A and YAP, indicating that YAP expression levels are positively correlated with Aurora A in lung cancer.

### Aurora A enhances the protein expression and transcription activity of YAP in lung cancer cells

To investigate the potential crosstalk between Aurora A and YAP in lung cancer cells, we depleted endogenous Aurora A and examined YAP protein expression. Aurora A knockdown attenuated YAP protein expression in A549 (Fig. 1d) and H1299 (Supplementary Fig. 1a) cells. Similar results were observed in the colorectal cancer cell line BGC (Supplementary Fig. 1b). However, in triple-negative breast cancer cell line MD-MBA-231 (Supplementary Fig. 1c), YAP protein expression is not affected when Aurora A is knocked down, which is in consistent with previously findings^23^. Besides, we also found in Her2^+^ breast cancer cell line SK-BR-3, knockdown of Aurora A could not change the protein level of YAP (Supplementary Fig. 1d). Therefore, the regulation of Aurora A on YAP protein expression varied in different cancer types. Moreover, Aurora A overexpression elevated the protein level of YAP (Fig. 1e). To further verify whether Aurora A regulates YAP specifically, we restored Aurora A expression in the Aurora A depleted cells, and found that the reduction of YAP expression was rescued in both A549 (Fig. 1f) and H1299 (Supplementary Fig. 1e) cells, indicating Aurora A acts as the upstream regulator of YAP.

Next, we examined the impact of Aurora A on the transcriptional activity of YAP using luciferase assay. The transcriptional activity of YAP was significantly reduced when Aurora A was knocked down both in A549 (Fig. 1g) and H1299 (Supplementary Fig. 1f) cells. Conversely, Aurora A overexpression dramatically enhanced YAP mediated-transcriptional activity (Supplementary Fig. 1g). Besides, Aurora A knockdown remarkably decreased the mRNA expression levels of the YAP targets *CTGF* and *CYR61* in A549 (Fig. 1h) and H1299 cells (Supplementary Fig. 1h), as well as the protein levels of CTGF in A549 cells (Fig. 1i). These results showed that Aurora A indeed enhances the protein expression and transcriptional activity of YAP.

### Kinase activity of Aurora A contributes to the regulation of YAP

**Fig. 2:**
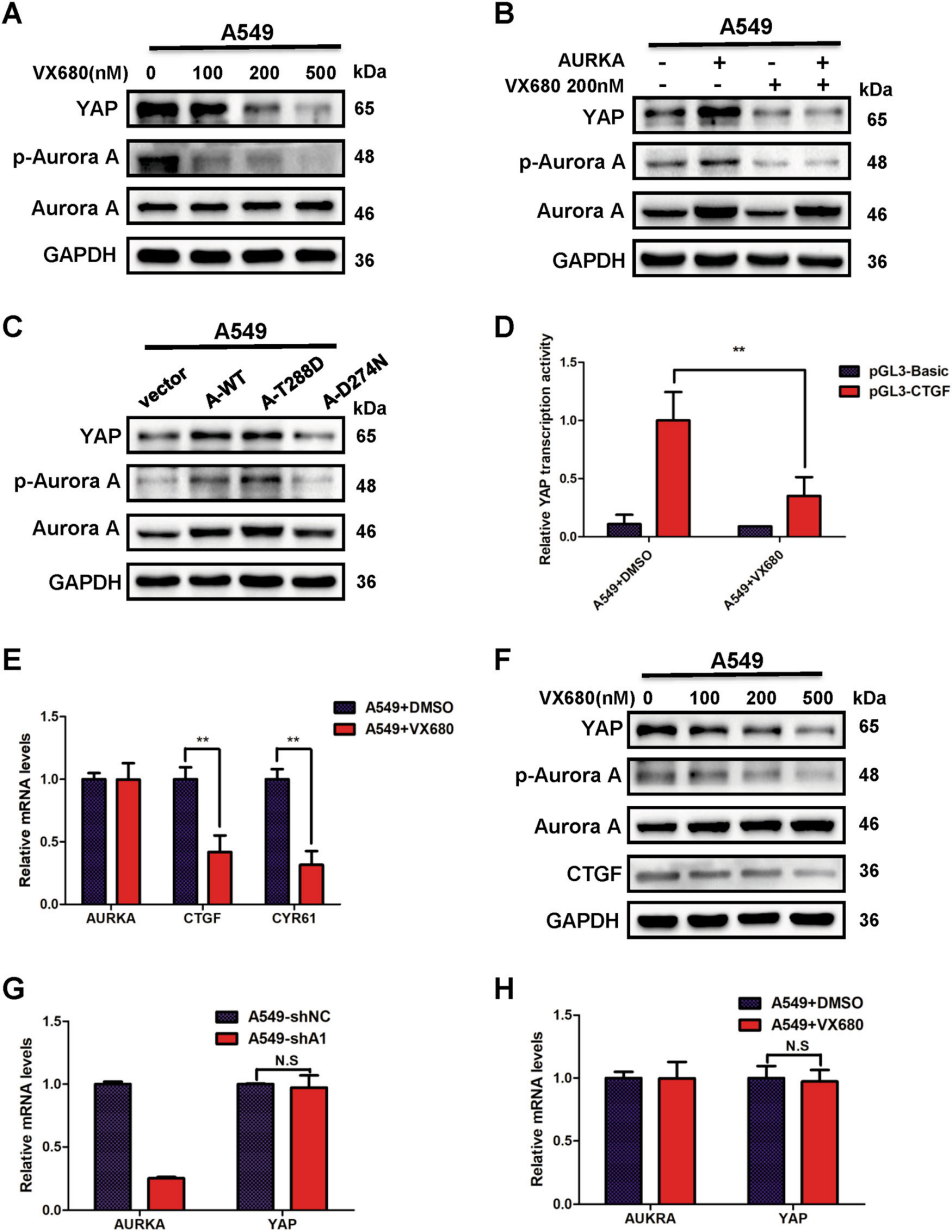
Aurora A regulates the protein expression and transcription activity of YAP through its kinase activity. **a** Western blot analysis of YAP protein level in A549 cells treated with indicated doses of VX-680 for 24 h. **b** Western blot analysis of YAP protein level in Aurora A overexpressed (AURKA) A549 cells incubated with VX-680 (200 nM) or DMSO for 24 h. **c** Western blot analysis of YAP protein level in A549 cells transfected with wild-type Aurora A(A-WT) and plasmids encoding different kinase forms of Aurora A (A-T288D, A-D274N) for 48 h. **d** Luciferase reporter assay to evaluate the activity of YAP from A549 cells treated with VX-680(200 nM) or DMSO for 24 h. Error bars represented mean ± S.D. (*n* = 3, **P < 0.01). **e** RT-qPCR analysis of the mRNA levels of YAP target genes in A549 cells treated with VX-680 (200 nM) or DMSO for 24 h. Error bars represented mean ± S.D. (*n* = 3, **P < 0.01). **f** Western blot analysis of the protein levels of YAP and its target gene CTGF in A549 cells treated with different doses of VX-680 for 24 h. **g** RT-qPCR analysis of YAP mRNA level in shAURKA (shA1) cells and control groups. Error bars represented mean ± S.D. (*n* = 3). **h** RT-qPCR analysis of YAP mRNA levels in A549 cells treated with VX-680 (200 nM) or DMSO. Error bars represented mean ± S.D. (*n* = 3)

A recent study has determined that YAP is a downstream substrate of Aurora A kinase in breast cancer^23^. Therefore, we want to investigate, in lung cancer, whether Aurora A stabilizes YAP protein expression through its kinase activity. We treated A549 cells with VX680, a kinase inhibitor of Aurora A in a dose-dependent manner. YAP protein level had an obvious decrease along with the increasing doses of VX680 in A549 (Fig. 2a). Conversely, Aurora A overexpression raised the protein level of YAP but this regulation could be blocked by VX680 (Fig. 2b). Moreover, we transfected A549 cells with wild type AURKA (AURKA-WT), constitutively active AURKA (AURKA-T288D) and kinase-dead AURKA(AURKA-D274N) plasmid, respectively. YAP was overexpressed in AURKA-WT and AURKA-T288D transfected cells; however, mutated Aurora A kinase (AURKA-D274N) that has no kinase activity failed to increase YAP expression (Fig. 2c).

The phosphorylation of Serine 397 is reported to be important for YAP subsequently translocating to cytoplasm for proteasomal degradation^11–13^. To figure out whether YAP protein stabilization promoted by Aurora A is dependent on nucleo/cytoplasmic translocation of YAP, we performed the nucleus and cytoplasm fractionation of the shAURKA and control A549 cells. The results showed that YAP protein level was reduced both in nucleus and cytoplasm, indicating that Aurora A could not induce the nucleo/cytoplasmic spread of YAP (Supplementary Fig. 2a). Furthermore, we constructed the mutant plasmid YAP S397A to transfect the Aurora A depleted or control cells. Unexpectedly, the mutation of S397A could not block the regulation of Aurora A on YAP in A549 and H1299 cells (Supplementary Fig. 2b). These results showed that the regulation of YAP by Aurora A is depending on the kinase activity but not through phosphorylating Serine 397 site of YAP in lung cancer.

Next, we used VX680 to inhibit Aurora A kinase activity and investigated the transcriptional activity of YAP. Compared with controls, YAP mediated-transcriptional activity was remarkably decreased when Aurora A kinase activity was inhibited by VX680 in A549 (Fig. 2d) and H1299 cells (Supplementary Fig. 2c). Moreover, the constitutively active Aurora A (AURKA-T288D) overexpression dramatically enhanced YAP mediated-transcriptional activity, but no effects on YAP transcription activity were observed in the cells expressing kinase-dead form of Aurora A (AURKA-D274N) (Supplementary Fig. 1g). In agreements, the mRNA expression levels of YAP targeted genes *CTGF* and *CYR61* (Fig. 2e, Supplementary Fig. 2d) were downregulated in the VX680 group compared with controls and the protein expression of CTGF was also decreased (Fig. 2f).

Collectively, our results suggested that Aurora A enhances the protein expression and transcriptional activity of YAP through its kinase activity.

### Aurora A has no influence on the mRNA expression of YAP

We next investigated if Aurora A regulated YAP mRNA expression in lung cancer cells. Aurora A knockdown did not attenuate YAP mRNA expression in A549 (Fig. 2g) and H1299 cells (Supplementary Fig. 2e). Similarly, when Aurora A kinase activity was inhibited, YAP mRNA levels did not decline in A549 (Fig. 2h) and H1299 (Supplementary Fig. 2f) cells, indicating that Aurora A does not repress YAP expression at the transcription level.

### Aurora A induces YAP protein expression independently of the Hippo pathway

**Fig. 3:**
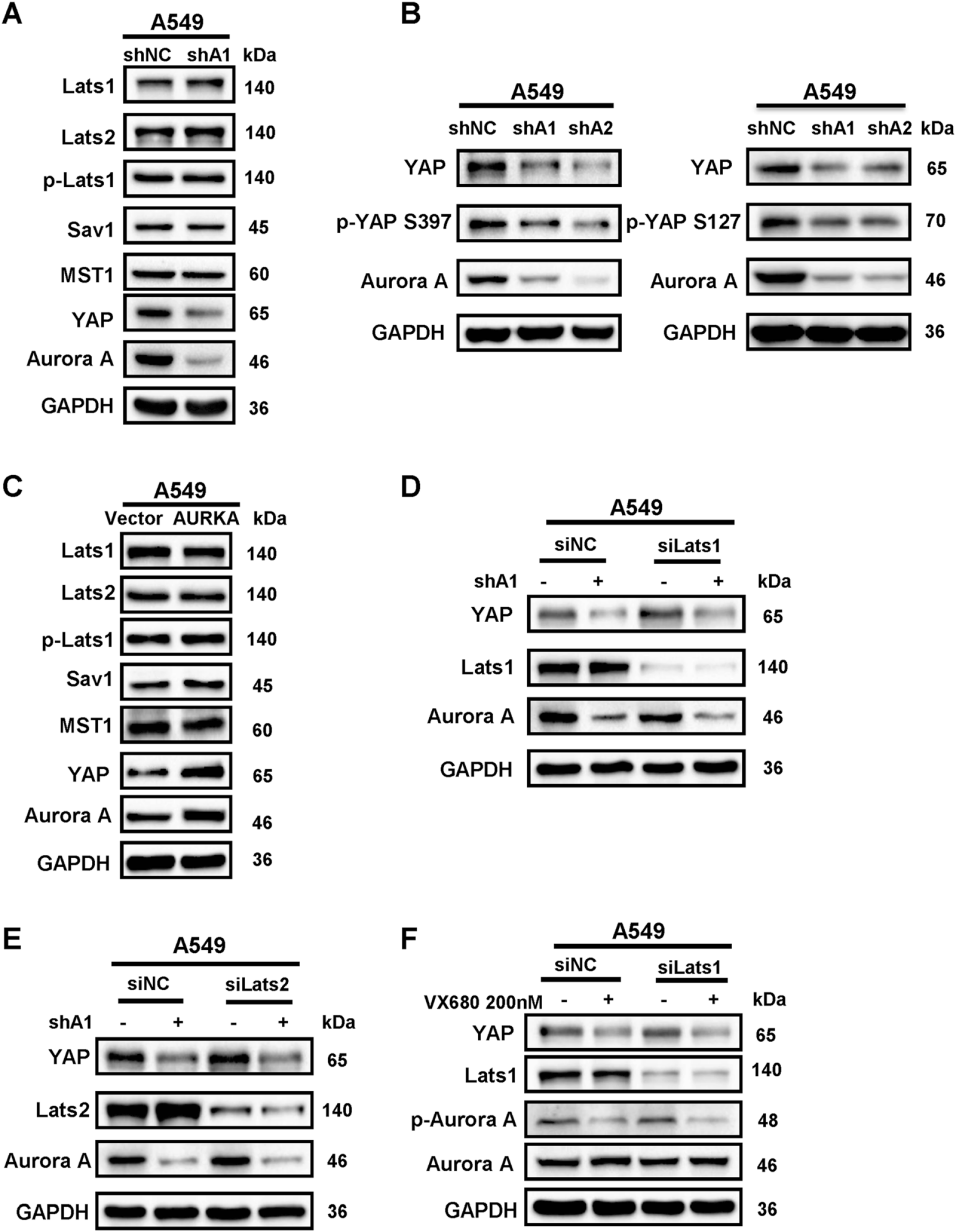
Aurora A stabilizes the protein level of YAP independently of the Hippo pathway. **a** Western blot analysis of the protein levels of key regulators in the Hippo pathway in A549 cells with Aurora A knockdown (shA1). **b** Western blot analysis of the protein levels of p-YAP (S397, S127) in A549 cells with Aurora A knockdown (shA1 or shA2). **c** Western blot analysis of the protein levels of key regulators in the Hippo pathway in A549 cells with Aurora A overexpression (AURKA). **d** Lats1 or **e** Lats2 were knocked down by siRNA in shAURKA (shA1) A549 cells, and YAP protein level was analyzed by western blot. **f** Lats1 was knocked down by siRNA in A549 cells, and then incubated with VX-680 (200 nM) or DMSO. YAP protein level was analyzed by western blot

In the Hippo signalling pathway, YAP protein level is known to be regulated by upstream kinases Lats1 and Lats2. Knockdown of Lats1 or Lats2 using small interfering RNA significantly decreased the phosphorylated forms of YAP (p-YAP S397 and p-YAP S127), and accordingly increased the total protein levels of YAP in A549 and H1299 cells (Supplementary Fig. 3a, b) as previously reported^11–13^. To probe whether Aurora A regulates YAP protein expression through Hippo pathway, we knocked down Aurora A and found that both the total YAP proteins and phosphorylated forms of YAP were reduced in A549 (Fig. 3a, b) and H1299 cells (Supplementary Fig. 3c), however the Lats1/2, Mst1/2 and SAV1 protein levels remained unchanged. Similar results were observed when Aurora A was overexpressed (Fig. 3c). Consistently, there were no changes in Lats1/2, Mst1/2 and SAV1 protein levels when Aurora A kinase activity was inhibited (Supplementary Fig. 3d). Furthermore, knockdown of Lats1 or Lats2 could not reverse the effects of Aurora A on YAP in A549 cells (Fig. 3d, e) and H1299 cells (Supplementary Fig. 3e). Similar results were observed when Aurora A kinase activity was inhibited by VX680 in A549 cells (Fig. 3f), confirming that Aurora A increases YAP protein abundance independently of the Hippo pathway.

### Aurora A depletion reduces the protein stability of YAP independently of the proteasome pathway

**Fig. 4:**
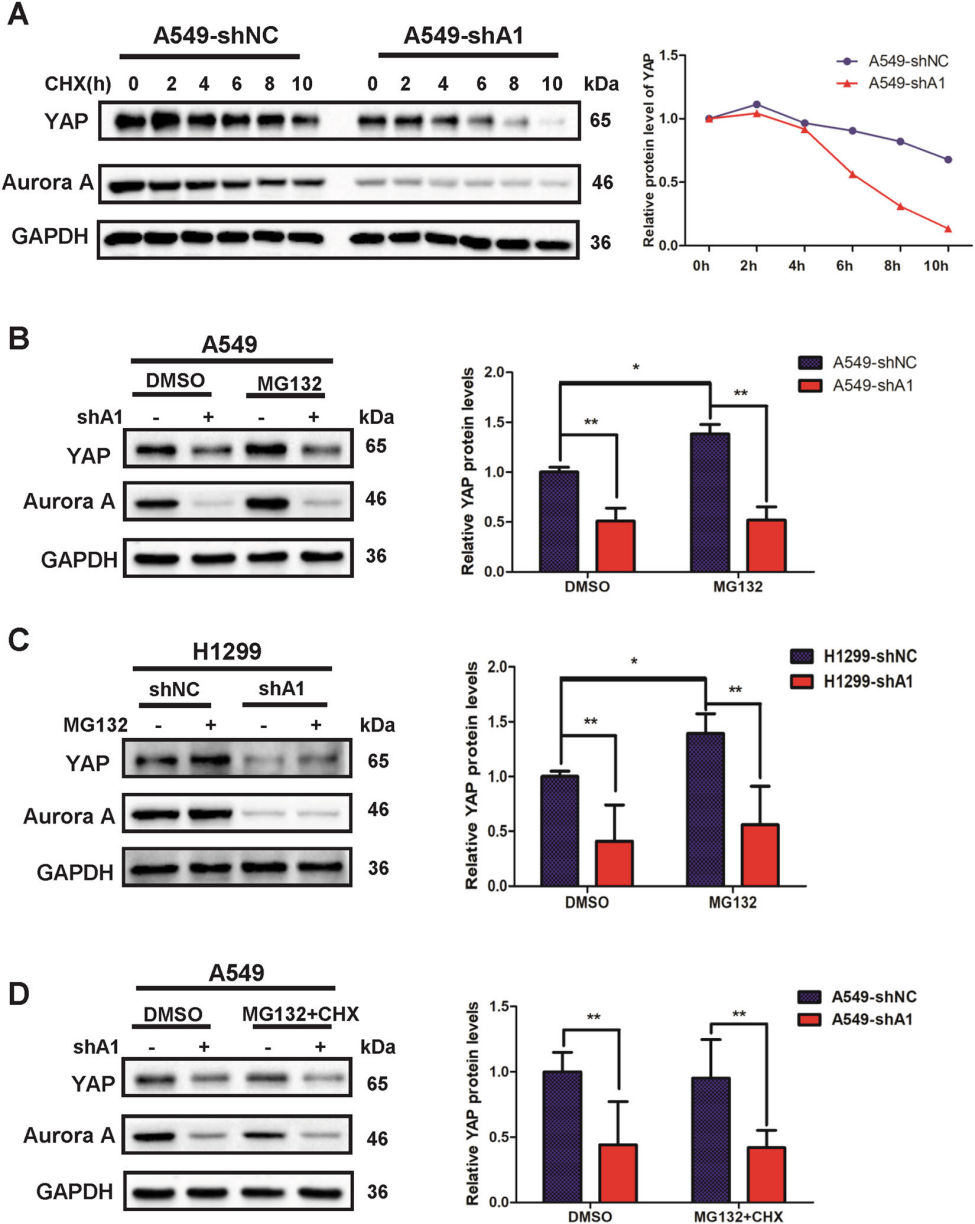
Aurora A knockdown reduces the protein stability of YAP independently of the proteasome pathway. **a** Aurora A depleted (shA1) and control groups (shNC) were treated with 100 μg/ml cycloheximide (CHX) for the indicated time and subjected for western blot analysis. The relative expression (right panel) are the ratios of YAP normalized to GAPDH. **b** Aurora A depleted (shA1) and control (shNC) A549 and **c** H1299 cells were treated with MG132 (10 μM) for 6 h, both Aurora A and YAP protein levels were analyzed by western blot. The relative expression shown (right panel) are mean ± S.D. of the ratios of YAP to GAPDH (*n* = 3, *P < 0.05; **P < 0.01). **d** Aurora A depleted (shA1) and control (shNC) groups were treated with CHX (100 μg/ml) and MG132 (10 μM) for 6 h and subjected for western blot analysis. The relative expression shown (right panel) are mean ± S.D. of the ratios of YAP to GAPDH (*n* = 3, **P < 0.01)

Our results led us to speculate that Aurora A may regulate YAP through modulating its protein stability. To test this conjecture, we treated Aurora A knockdown or control cells with cycloheximide (CHX) to inhibit de novo protein synthesis. Aurora A depletion dramatically reduced YAP protein half-life (Fig. 4a), indicating that Aurora A stabilizes YAP and increases protein abundance at the post-translational level. To confirm whether the decrease of YAP expression was mediated by proteasomal degradation, we treated the cells with the proteasome inhibitor, MG132. YAP protein expression could not be rescued by MG132 treatment in Aurora A knockdown A549 and H1299 cells (Fig. 4b, c). Conversely, in the control cells, YAP protein levels were enhanced by MG132 treatment. The addition of MG132 along with CHX also could not block the modulation of YAP expression by Aurora A (Fig. 4d). These results led us to conclude that Aurora A induces the protein stability of YAP independently of the proteasome pathway.

### Aurora A increases YAP protein abundance through the blockage of autophagy

**Fig. 5:**
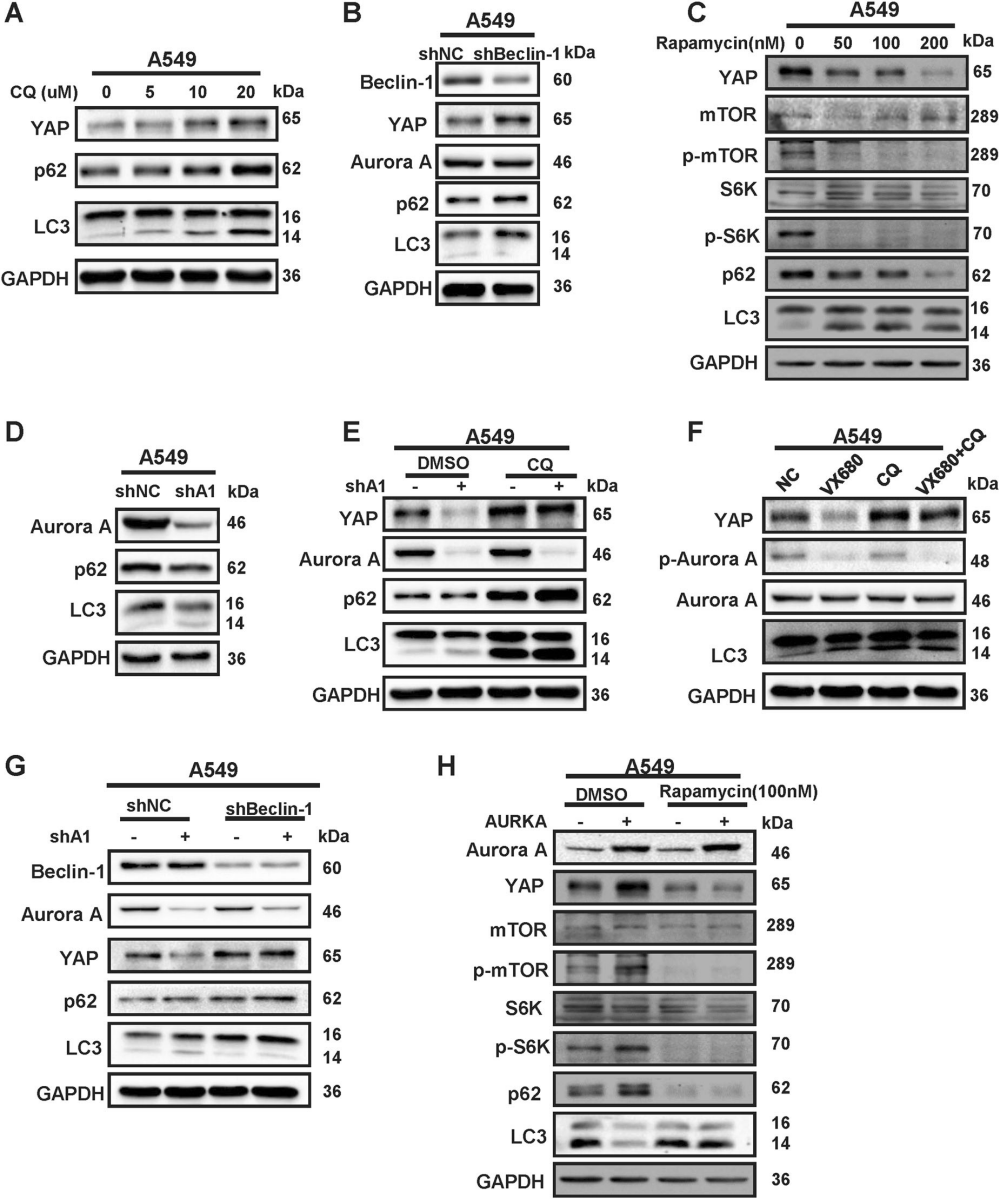
Aurora A induces the protein expression of YAP through suppressing autophagy. **a** Western blot analysis of YAP, p62 and LC3 proteins level in A549 cells incubated with different doses of CQ for 12 h. **b** Western blot analysis of YAP, p62 and LC3 proteins level in A549 cells with Beclin-1 depletion. **c** A549 cells were treated with vehicle control or indicated concentration of rapamycin for 24 h followed by western blot analysis with indicated antibodies. **d** Western blot analysis of p62 and LC3 protein levels in A549 cells with Aurora A depletion (shA1). **e** Aurora A depleted (shA1) and control (shNC) cells were treated with or without chloroquine (CQ) for 12 h and subjected to western blot analysis with indicated antibodies. **f** A549 cells were treated with VX-680, with or without CQ for 12 h and subjected to western blot. **g** Beclin-1 was knocked down in Aurora A depleted and control cells, and indicated proteins were analyzed by western blot. **h** Expression of the indicated proteins was analyzed by western blot in Aurora A overexpressed and control cells treated with or without rapamycin (100 nM) for 24 h

Our previous studies showed that Aurora A blocks the autophagy through activating the mTOR pathway in breast cancer^36,37^. We next investigated if Aurora A promotes YAP protein stability via inhibiting autophagy, which is a lysosome-dependent protein degradation pathway. To this end, we used the lysosomotropic and autophagy inhibitor chloroquine (CQ) to block the autophagy-induced protein degradation. As expected, the YAP protein level increased dramatically upon CQ treatment in a dose-dependent manner (Fig. 5a). SQSTM/p62 is a well-known ubiquitin- and LC3-binding protein which expression level increases when autophagy is impaired, while LC3-II is the faster migrating species of the cleaved LC3 and its accumulation is caused by blockage of autophagosome^38^. Therefore, we introduced the markers p62 and LC3 of autophagy in western blot analysis. As shown in the Fig. 5a, CQ treatment caused the accumulation of these markers, indicating the involvement of autophagy. Next, we used shRNA to deplete the autophagosome-initiating Beclin-1 complex. In agreement, the protein expression of YAP was induced by Beclin-1 knockdown (Fig. 5b). It is newly reported that YAP accumulation is due to impaired degradation of the protein by the autophagosome/lysosome system^46^ and our previous study confirmed that Aurora A blocks the autophagy through activating the mTOR pathway^37^. Therefore, we used rapamycin, a mTOR inhibitor, to promote autophagosome formation and autophagy. As shown in Fig. 5c, the phosphorylated form of mTOR and S6K were reduced, and there was a dramatic reduction in protein levels of YAP upon administration of the mTOR inhibitor. These data indicated that YAP is a cargo of autophagy-induced protein degradation.

To confirm that Aurora A promotes YAP stability through blocking autophagy, we firstly verified that Aurora A depletion reduced p62 and increased LC3-II expression and therefore, enhanced autophagy (Fig. 5d). Then we examined the expression levels of YAP following Aurora A depletion in the absence or presence of CQ and found that Aurora A knockdown reduced YAP expression only in the control but not in the CQ-treated cells (Fig. 5e). In agreement, A549 cells co-incubated with CQ, showed no reduction in YAP protein expression upon treatment with the Aurora A kinase inhibitor VX680 (Fig. 5f). Furthermore, YAP expression was only downregulated in response to Aurora A knockdown in control but not in Beclin-1 depleted A549 cells (Fig. 5g). And we also used rapamycin to inhibit mTOR activity and found that the phosphorylated form of mTOR and S6K decreased but Aurora A overexpression was unable to increase YAP expression in the presence of rapamycin (Fig. 5h). These data suggested that Aurora A protects the protein stability of YAP at least partially through inducing the activity of mTOR which suppresses the autophagy.

### YAP acts as a downstream factor of Aurora A to promote the proliferation and migration of lung cancer cells

**Fig. 6:**
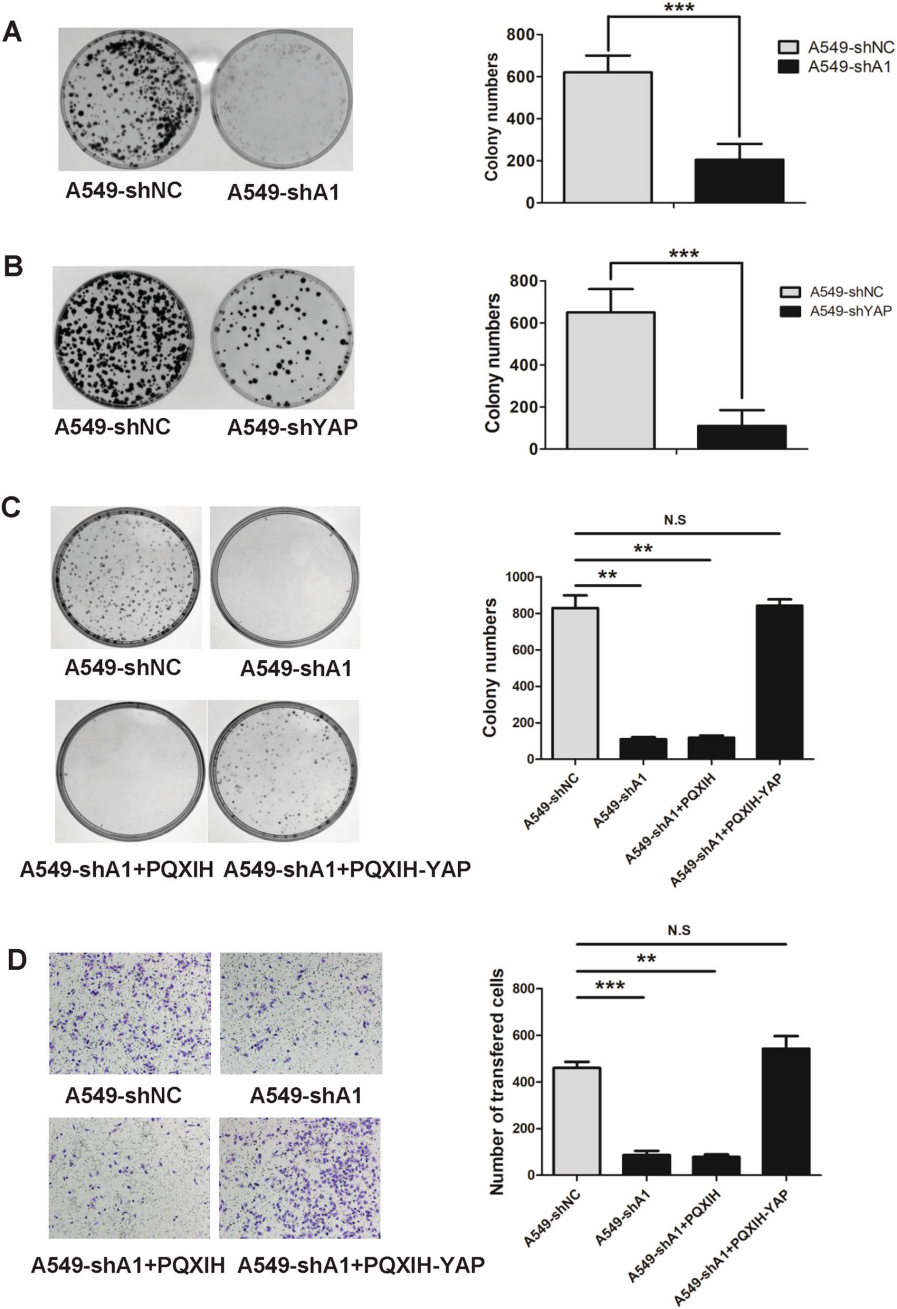
YAP acts as a downstream factor of Aurora A to promote the proliferation and migration of lung cancer cells. **a** Colony formation abilities analysis of Aurora A depleted (shA1) and control (shNC) cells. **b** Colony formation abilities analysis of YAP depleted(shYAP) and control cells. **c** Colony formation abilities analysis of Aurora A depleted (shA1) and control(shNC), Aurora A depleted with YAP overexpressed (shA1 + PQXIH-YAP) and control (shA1 + PQXIH) groups. **d** Migration abilities analysis of Aurora A depleted(shA1) and control (shNC), and Aurora A depleted with YAP overexpressed (shA1 + PQXIH-YAP) and control (shA1 + PQXIH) groups. Static analysis was shown. Error bars represented mean ± S.D. (*n* = 3, *P < 0.05; **P < 0.01 and ***P < 0.001)

To assess the contribution of Aurora A and YAP to lung cancer cell proliferation and migration, we knocked down Aurora A and YAP independently in the A549 and H1299 lung cancer cell lines. Both Aurora A and YAP knockdown significantly inhibited lung cancer cell proliferation (Fig. 6a, b, Supplementary Fig. 4a, b) and migration (Supplementary Fig. 4c, d). Conversely, ectopic expression of Aurora A contributed to an increase in proliferation and migration in lung cancer cells (Supplementary Fig. 5a, b). Similarly, ectopic expression of YAP also resulted in an increase in proliferation and migration rates in lung cancer cells (Supplementary Fig. 5c, d). Moreover, Aurora A depletion suppressed lung cancer cell colony formation and migration ability, which could be reversed by YAP ectopic overexpression (Fig. 6c, d). Taken together, these findings suggest that YAP functions downstream of Aurora A to promote the proliferation and migration of lung cancer cells.

## Discussion

As an oncogene, YAP protein expression level is canonically determined by the LATS kinase of Hippo pathway^13^, and important for tumor initiation^47^, progression^14^, metastasis^48^ and immune evasion^49^. However, the regulatory mechanisms of YAP stability independent of the Hippo pathway have hitherto not been elucidated. In this study, we discover for the first time that Aurora A is a critical regulator in stabilizing YAP protein expression in lung cancer. Mechanically, Aurora A increases YAP protein abundance due to the blockage of autophagy, and this regulation is dependent of Aurora A kinase activity. Moreover, YAP expression levels are positively correlated with Aurora A in lung cancer. These findings demonstrate a previously unrecognized but critical pathway for YAP stability regulation and the important role of Aurora A-YAP axis in the progression of lung cancer.

YAP stability is regulated by LATS kinase in a proteasome-dependent manner^11–13^. Specifically, the phosphorylation of YAP at Serine 397 by LATS facilitates YAP for subsequent poly-ubiquitination and degradation. As an oncogene, Aurora-A plays multiple roles in regulating cancer development *via* promoting cell cycle progression, activating cell survival and/or anti-apoptosis signaling, enhancing tumorigenicity of oncogenes, and contributing to epithelial-mesenchymal transition (EMT) and stem-like properties of cancer cells^39^. Herein, we demonstrate that Aurora A stabilizes YAP protein level in lung cancer. However, Aurora A depletion does not affect the LATS kinase, the known upstream regulators of YAP in the Hippo pathway. Moreover, we reveal that Aurora A regulation on YAP is dependent of its kinase activity. Recently, YAP is reported to be phosphorylated by Aurora A at the same site Serine 397 in triple-negative breast cancer cell lines^23^. We then constructed the mutation site S397A and found that the mutation of S397A could not block the regulation of Aurora A on YAP in lung cancer. Accordingly, the reduce of the phosphorylated YAP protein level was probably due to the decrease of YAP protein level in A549 cells with Aurora A depletion. These results indicate that the regulation of YAP by Aurora A is depending on the kinase activity and possibly through unrecognized mechanisms for promoting YAP protein stability.

Autophagy is a digestive pathway that captures, degrades, and recycles intracellular proteins and organelles in lysosomes and promotes survival^50^. Evidence has suggested that autophagy inhibition may be therapeutically advantageous for cancer therapy^51,52^. Autophagy is negatively regulated by a number of kinases including mTOR, and our previous studies have proposed that depletion or inhibition of Aurora A decreases phosphorylated mTOR expression under starvation conditions and thus activates autophagy^36,53^. In here, we reveal that Aurora A protects YAP protein stability via inhibiting autophagy. We used the CQ to prevent the protein degradation induced by autophagy, and knocking down Beclin-1 to inhibit the autophagy-specific class III PI3K complex which is essential for autophagy. When autophagy was blocked by CQ or Beclin-1 knockdown, the expression of YAP was substantially elevated. Conversely, when autophagy was enhanced by treating the cells with rapamycin, the protein levels of YAP were reduced significantly. Moreover, no decrease of YAP protein level was found in Aurora A knockdown cells, when the autophagy was inhibited by CQ and upon Beclin-1 knockdown. Similarly, YAP expression was not increased by Aurora A overexpression when mTOR activity was inhibited by rapamycin. As YAP is reported to activate mTOR by inducing miR-29 to inhibit PTEN translation^54^, there may be a positive feedback loop between YAP and mTOR reciprocal regulation. More studies to delineate this feedback loop are of future interest. Collectively, these results show that Aurora A is unable to regulate YAP expression when autophagy is blocked, suggesting that Aurora A regulates YAP expression through repressing autophagy.

Inhibiting YAP/TAZ activity offers an attractive anti-cancer strategy, however, it is challenging to design direct activators for protein kinases and inhibitors for transcriptional co-factors. Indeed, porphyrin family molecules, especially verteporfin, can disrupt the interaction between YAP/TAZ and TEADs, thus suppressing tumor growth in mouse models^55^. However, verteporfin has general cellular toxicity and low aqueous solubility. As an oncogene, Aurora A overexpresses in many tumors, and Aurora A kinase inhibitors have been approved in clinical trials and achieved promising therapeutic effects^56^. Herein, we demonstrated that Aurora A and YAP expression levels are highly correlated in lung cancer tissues, and YAP is a critical downstream effector of Aurora A in lung cancer development. YAP protein level and YAP mediated-transcriptional activity was remarkably decreased when Aurora A or its kinase activity was inhibited. Therefore, targeting Aurora A may be an accessible strategy for cancers that highly express YAP.

In summary, we demonstrate here for the first time that activation of Aurora A kinase increases YAP stability via blockage of autophagy, a novel mechanism for the regulation of the oncogene YAP in lung cancer. The Aurora A-YAP axis could be a potential promising therapeutic target for cancer intervention and therapy.

## Materials and methods

### Cell lines and cell culture

The human lung cancer cell lines (A549, H1299), breast cancer cell lines (MD-MBA-231, SK-BR-3) and colon cancer cell line BGC were obtained from the American Type Culture Collection (ATCC). A549, H1299 and BGC cells were routinely maintained in RPMI 1640 medium (Invitrogen) supplemented with 10% fetal bovine serum (FBS, HyClone). MD-MBA-231 and SK-BR-3 cells were cultured in DMEM medium (Invitrogen) supplemented with 10% fetal bovine serum (FBS, HyClone). Treatments with doxycycline (Clonetech), VX-680 (Selleck Chemicals), cycloheximide (Amresco) and MG132 (Sigma Aldrich) were carried out as indicated.

### Western blot analysis

Cellular or tissue samples were lysed with RIPA lysis buffer freshly adding cocktail protease inhibitor (Thermo Scientific). Equal amounts of cellular proteins were subjected to electrophoresis in SDS-PAGE, and transferred to nitrocellulose membranes (Millipore). The membranes were blocked and then incubated at 4 °C overnight with indicated first antibodies, followed by incubation with the appropriate HRP-conjugated secondary antibodies (Thermo Scientific). The protein bands were detected and analyzed with an ECL. The following antibodies were used: anti-Aurora A (Upstate, #07-648), anti-phospho-Aurora A (Thr288) (Cell Signaling Technology, #3079), anti-YAP (Cell Signaling Technology, #4912), anti-phospho-YAP(ser127) (Cell Signaling Technology, #4911), anti-phospho-YAP(Ser397) (Cell Signaling Technology, #13619), anti-Lats1 (Cell Signaling Technology, #3477 P), anti-phospho-Lats1 (Thr1079) (Cell Signaling Technology, #9654P), anti-Lats2 (Rui Ying biological, China, #RLP1047), anti-SAV1 (Cell Signaling Technology, #3507P), anti-MST1 (Cell Signaling Technology, #3682P), anti-GAPDH (Kangcheng, China,#KC-5G4), anti-Lamin B1 (Epitomics, #6581-1), anti-CTGF (Life Science Products & Services, #AB60212a), anti-Myc Tag (MERCK, 05-724).

### Immunohistochemistry assay

The resected specimens were obtained from primary lesions, fixed with formalin, embedded with paraffins. Serial 4 μm sections were prepared, then briefly incubated with xylene, rehydrated with graded ethanol solutions, incubated with methyl alcohol containing 3% hydrogen peroxide and immersed in a citrate buffer for antigen retrieval. IHC was performed using Streptavidin-Peroxidase IHC assay kit (ZSGB-bio, China) following the manufacturer’s instructions. Antibodies of Aurora A and YAP diluted 1:200 in PBS containing 2% goat bovine serum respectively. Immunostaining was evaluated by two pulmonary pathologists using a blind protocol design. For each specimen, the total score of intensity expression (negative staining: 0 point; weak staining: 1 point; moderate staining: 2 point; and strong staining: 3 point) and multiplying stained cell numbers (positive cells as ≤25% of the cells: 1 point; 26–50% of the cells: 2 points; 51–75% of the cells: 3 points; >75% of the cells: 4 points) of Aurora A or YAP was estimated. When the sample was scored ≥ 6 points, we defined it as high expression, otherwise low expression.

### RNA extraction and quantitative RT-PCR (RT-qPCR) assays

Total RNA was extracted by using TRIzol reagent (Life Technologies, 15596026). The 300 EasyScript One-Step gDNA Removal and cDNA Synthesis SuperMix kit 301 (TransGene Biotech, #AE311-03) were used to generate cDNA. RT-qPCR was performed by using Platinum SYBER green PCR supermix (Invitrogen) and the Mx3005P QPCR System (Agilent Technologies). Primers were purchased from Invitrogen (Shanghai, China) and primer sequences are as follows: AURKA, forward: 5′-CCACCTTCGGCATCCTAATA-3′ and reverse:

5′-TCCAAGTGGTGCATATTCCA-3′. YAP, forward: 5′-CCTCGTTTTGCCATGAACCAG-3′ and reverse: 5′-GTTCTTGCTGTTTCAGCCGCAG-3′. CTGF:forward: 5′-TGCCCTCGCGGCTTACCGACTG-3′ and reverse: 5′-TGCAGGAGGCGTTGTCATTGGTAAC-3′.CYR61, forward: 5′-GGTCAAAGTTACCGGGCAGT-3′ and reverse: 5′-GGAGGCATCGAATCCCAGC-3′. GAPDH, forward: 5′-GAAGGTGAAGGTCGGAGTC-3′ and reverse: 5′-GAAGATGGTGATGGGATTTC-3′. Changes of mRNA levels were determined by the 2^−△△CT^ method using GAPDH for internal crossing normalization.

### Plasmids, transfections and lentiviral packaging

shRNA-expressing vectors were constructed from the tet-pLKO-puro (Addgene) plasmid. The shRNA sequence are as follows: shAURKA1, 5′-GCACCACTTGGAACAGTTTAT-3′; shAURKA2, 5′-CCCUGUCUUACUGUCA-3′; shYAP, 5′-GCCACCAAGCTAGATAAAGAA-3′; shGFP, 5′-GCAAGCTGACCCTGAAGTTCAT-3′; Human full-length YAP plasmid PQXIH-YAP and empty PQXIH were kind gifts from Bin Zhao (Zhejiang University). The PQXIH-YAPS397A-myc plasmid primer sequence are as follows: Yap-S397A-F, 5′-AGATGAGgctACAGACAGTGGACTAAGCATGAGC-3′; Yap-S397A-R, 5′-TGTCTGTagcCTCATCTCGAGAGTGATAGGTGCC-3′. Plvx-vector and plvx-AURKA plasmids encoding different kinase forms of Aurora A were constructed as previously described^37^. All plasmids were verified by sequencing. Cells were transiently transfected using the lipofectamine 2000 (Invitrogen) with varying amounts of plasmid DNA. The siRNA sequence are as follows: siLats1, sense: 5′-AUUCGGGAAUCCCUUAGGA-3′, and antisense: 5′-UCCUAAGGGAUUCCCGAAUTT-3′; siLats2, sense: 5′-CAAGCAUCCUGAGCACGCA-3′, and antisense: 5′-UGCGUGCUCAGGAUGCUUGTT-3′; Negative control, sense: 5′-UUCUCCGAACGUGUCACGUTT-3′ and antisense: 5′-ACGUGACACGUUCGGAGAATT-3′ Lentiviral packaging was performed in HEK293T cells by co-transfecting lentiviral construct plasmid with psPAX2 (Addgene) and pMD2.G (Addgene), and stable clones were selected using puromycine (Sigma).

### Promoter assay

Transient cells co-transfections of the promoter-reporters were performed in 12 well dishes. The pGL3-basic (Promega, USA) was used as control promoter and pRSV-Renilla luciferase expression vector (Promega, USA) was used to monitor transfection efficiency. Cells were transfected with *CTGF* promoter-driven luciferase constructs (pGL3-*CTGF*) or control (pGL3-Basic) luciferase constructs using Lipofectamine2000 according to the manufacturer’s instruction. After 24 h of transfection, cells were lysed in passive lysis buffer and dual luciferase assays (Promega, USA) performed as manufacturer’s instructions. The Firefly luciferase activity was normalized to Renilla levels and is shown relative to control conditions.

### Colony formation assay

Approximately 1000 cells were plated into 60 mm dishes and cultured at 37 °C equipped with 5% CO_2_. Cells were cultured with RPMI 1640 medium supplemented with 10% fetal bovine serum every 3 days. After 14 days of incubation, colonies were fixed with 4% PFA, stained with crystal violet, and counted using Image J software. Each experiment was repeated three times.

### Transwell migration assays

Cells (5 × 10^4^) were placed into the upper chamber (24-well insert, 8 μm, Corning Costar) with FBS-free RPMI 1640 medium. RPMI 1640 medium supplemented with 10% FBS was used as an attractant in the lower chamber. After being incubated for 36 h, cells migrated through the membrane were fixed with 4% paraformaldehyde (Santa Cruz) and stained with 1% crystal violet (Shanghai Sangon Company). The stained cell images were captured by microscope (Olympus), and five random fields at ×10 magnification were counted. Results represented the average of triplicate samples from three independent experiments.

### Statistics

Each experiment was performed at least three times. Statistical tests were performed using SPSS Statistical 17.0. An unpaired Student’s *t* test was used for statistical comparisons between two groups. One-way ANOVA followed by the least significant difference test was used for multiple comparisons. The Kruskal-Wallis test was used for statistical comparisons of data with non-normal distributions. All data are expressed as the mean ± standard error (S.D.). *P < 0.05; **P < 0.01; ***P < 0.001.

## Supplementary information

Supplementary Information

Supplementary Figure 1

Supplementary Figure 2

Supplementary Figure 3

Supplementary Figure 4

Supplementary Figure 5

Supplementary Table 1
