# Supplementary material for: Activation of Aurora A kinase increases YAP stability via blockage of autophagy
Source: Cell Death Dis. 2019 Jun 3;10(6):432. doi: 10.1038/s41419-019-1664-4 (PMC6547697; doi:10.1038/s41419-019-1664-4)
Supplement: Supplementary file 1 — Supplementary Information [file 41419_2019_1664_MOESM1_ESM.pdf]

Supplementary Figure 1 Aurora A regulates the protein expression and transcription activity of YAP. (A) Western blot analysis of YAP protein expression in H1299 cells with Aurora A knockdown (shA1 or shA2). **(**B) Western blot analysis of YAP protein expression in BGC cells with Aurora A knockdown(shA1). (C)Western blot analysis of YAP protein expression in MDA-MB-231 or (D) SK-BR-3 cells with Aurora A knockdown (shA1 or shA2). (E) Aurora A knockdown H1299 cells (shA1) were transfected with plvx or plvx-AURKA plasmids. Western blot analysis of YAP protein expression. (F) Luciferase reporter assay to evaluate the activity of YAP from H1299 cells with Aurora A knockdown(shA1). Error bars represented mean ± S.D. (n=3, **P<0.01). (G) Luciferase reporter assay to evaluate the activity of YAP from A549 cells transfected with wild-type Aurora A(A-WT) or plasmids encoding different kinase forms of Aurora A (A-T288D, A-D274N). Error bars represented mean ± S.D. (n=3, **P<0.01, ***P<0.001). (H) RT-qPCR analysis of YAP mRNA level in H1299 cells with Aurora A knockdown(shA1). Error bars represented mean ± S.D. (n=3, **P<0.01)

Supplementary Figure 2 Aurora A regulates the protein expression and transcription activity of YAP through its kinase activity. (A) Western blot analysis of YAP and Aurora A protein level in cytoplasm or nucleus in Aurora A knockdown(shA1) or control A549 cells. Lamin B1 and GAPDH were taken as nuclear and cytoplasmic control respectively. Statistical analysis of relative ratio of nuclear to cytoplasmic YAP proteins levels. Error bars represented mean ± S.D.(n=3). (B) Western blot analysis of YAP protein level in PQXIH-YAPS397A-myc expressing (a) A549 and (b) H1299 cells with Aurora A knockdown(shA1). (C) Luciferase reporter assay to evaluate the activity of YAP from H1299 cells incubated with VX-680 (200 nM) or DMSO. Error bars represented mean ± S.D. (n=3, **P<0.01). (D) RT-qPCR analysis of YAP target genes mRNA level from H1299 cells treated with VX-680 (200nM) or DMSO. Error bars represented mean ± S.D. (n=3, **P<0.01).(E) RT-qPCR analysis of YAP mRNA level in H1299 cells with Aurora A knockdown(shA1). Error bars represented mean ± S.D. (n=3) (F) RT-qPCR analysis of YAP mRNA level from H1299 cells treated with VX-680 (200nM)

or DMSO. Error bars represented mean ± S.D. (n=3).

Supplementary Figure 3 Aurora A stabilizes the protein level of YAP independently of the Hippo pathway. (A) Lats1 or Lats2 was knocked down by siRNA in A549 or (B) H1299cells, YAP and phosphorylated YAP (p-YAP S127, p-YAP S397) levels were detected by western blot. (C) Western blot analysis of p-YAP S127 and p-YAP S397 in H1299 cells with Aurora A knockdown (shA1or shA2). (D) The protein levels of YAP and its key regulators in the Hippo pathway were detected by western blot in H1299 cells treated with different doses of VX-680. (E) Lats1 was knocked down by siRNA in H1299 cells with Aurora A knockdown(shA1), YAP protein levels were detected by western blot.

Supplementary Figure 4 Knockdown of Aurora A or YAP suppresses the proliferation and migration of lung cancer cells. (A) Western blot analysis of the knockdown or overexpression efficiency of Aurora A or YAP in A549 and H1299 cells. (B)Colony formation abilities analysis of H1299 cells with (a)Aurora A knockdown(shA1) or (b)YAP knockdown(shYAP). (C) Migration abilities analysis of A549 cells with (a)Aurora A knockdown(shA1) or (b)YAP knockdown(shYAP). (D) Migration abilities analysis of H1299 cells with (a)Aurora A knockdown(shA1) or (b) YAP knockdown(shYAP). Static analysis was shown. Error bars represented mean ± S.D. (n=3, **P<0.01, ***P<0.001).

Supplementary Figure 5 Ectopic overexpression of Aurora A or YAP promotes the proliferation and migration of lung cancer cells. (A) Colony formation abilities analysis of (a) A549 cells or (b) H1299 cells with Aurora A overexpression(AURKA). (B) Migration abilities measurement of (a)A549 or (b) H1299 cells with Aurora A (AURKA) overexpression by transwell assay. (C) Colony formation abilities analysis of (a) A549 or (b) H1299 cells with YAP overexpression(YAP). (D) Migration abilities measurement of (a)A549 or (b) H1299 cells with YAP overexpressed (YAP) by transwell assay. Static analysis was shown. Error bars represented mean ± S.D. (n=3, **P<0.01, ***P<0.001).
