# Supplementary figures and images for: Activation of Aurora A kinase increases YAP stability via blockage of autophagy
Source: Cell Death Dis. 2019 Jun 3;10(6):432. doi: 10.1038/s41419-019-1664-4 (PMC6547697; doi:10.1038/s41419-019-1664-4)

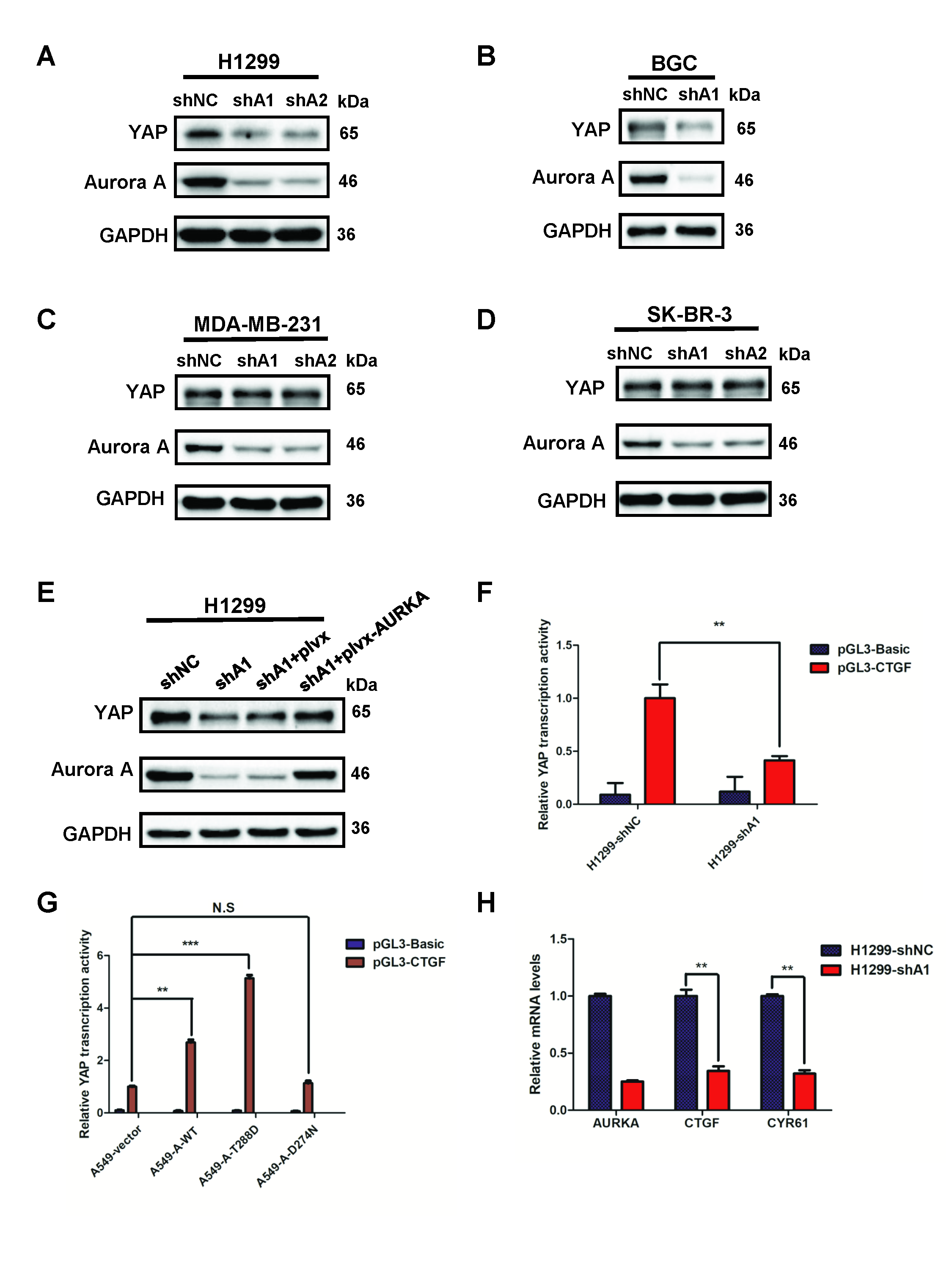

Supplement: Supplementary file 2 — Supplementary Figure 1 [file 41419_2019_1664_MOESM2_ESM.tif]

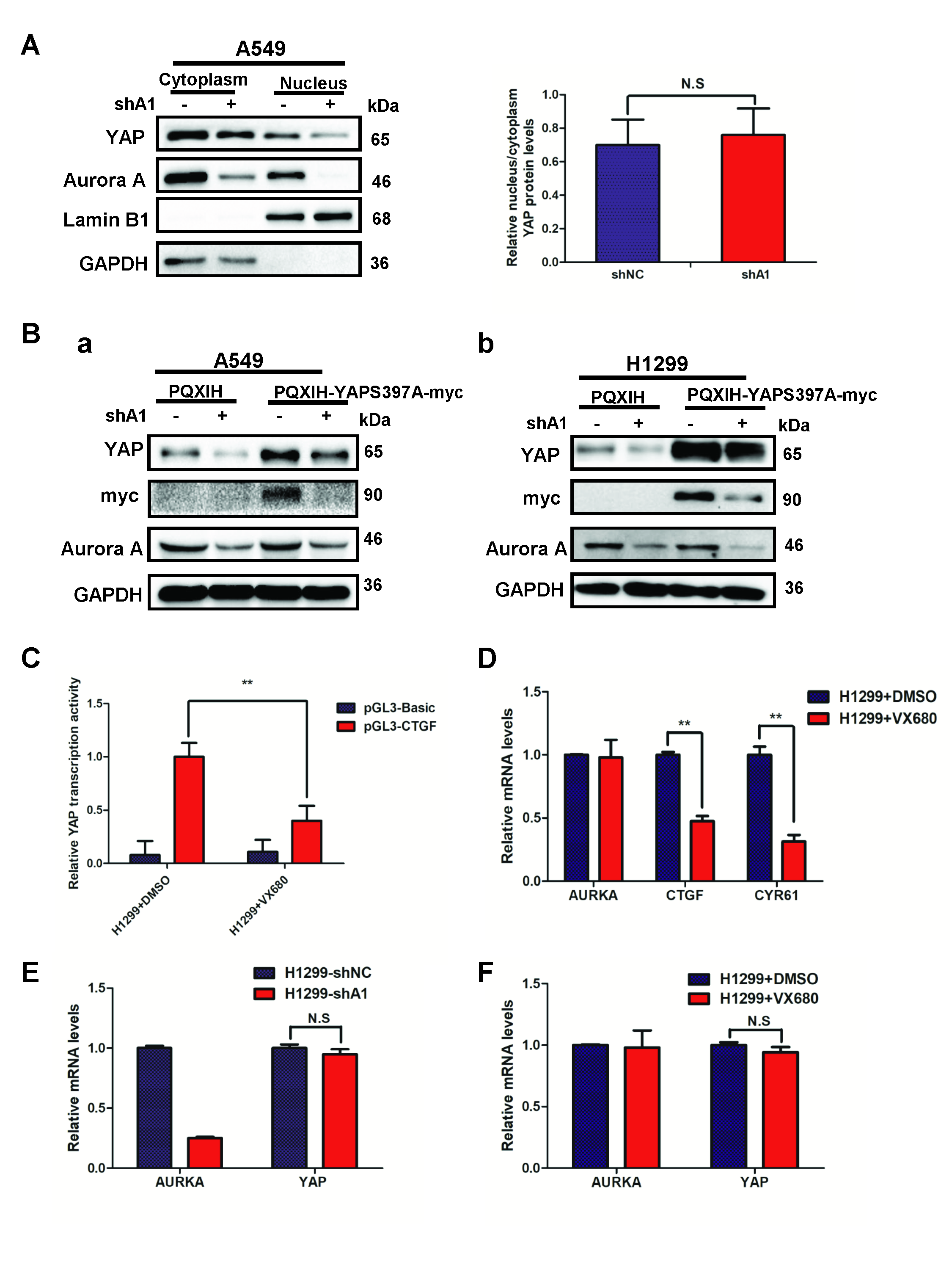

Supplement: Supplementary file 3 — Supplementary Figure 2 [file 41419_2019_1664_MOESM3_ESM.tif]

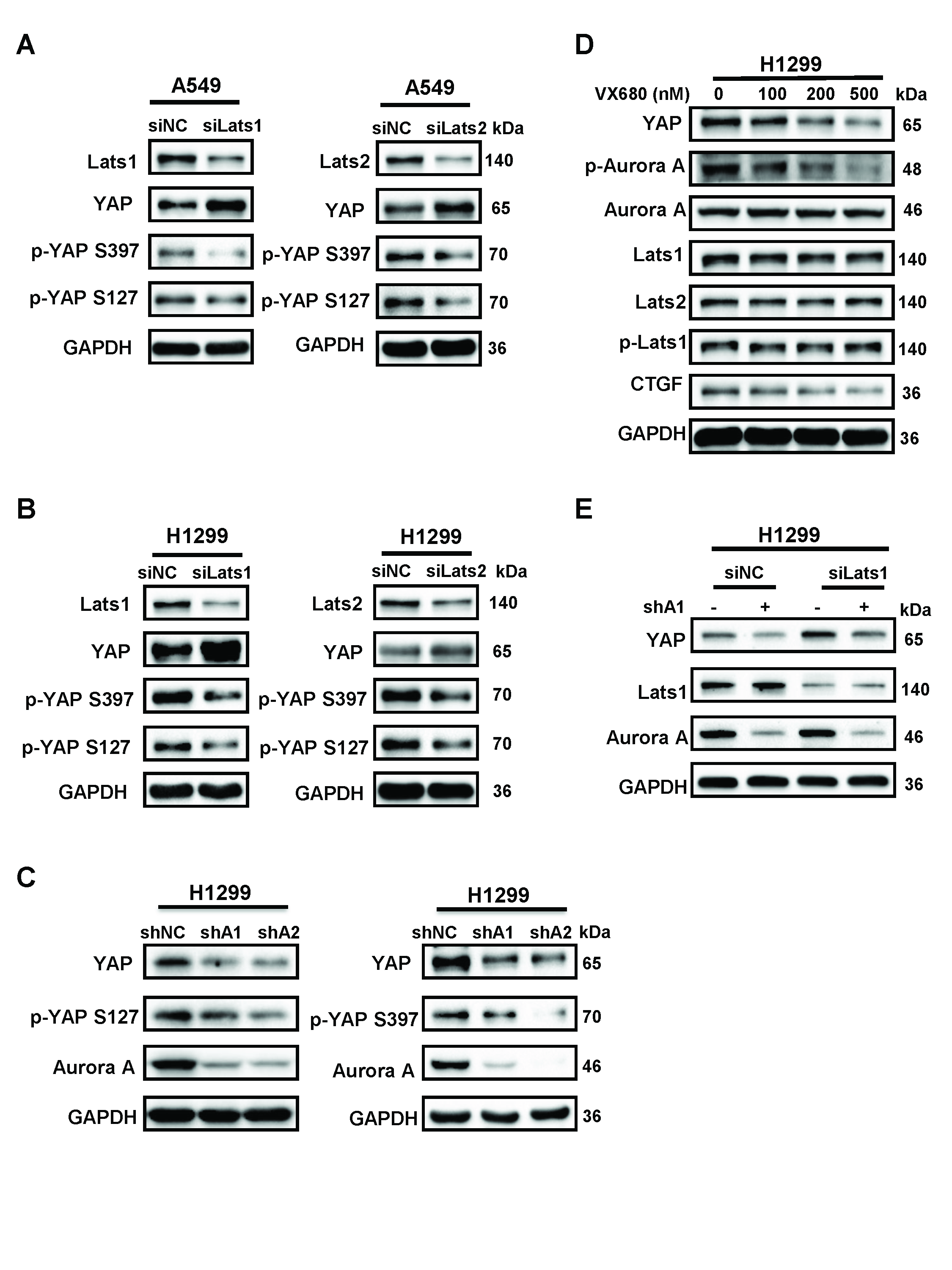

Supplement: Supplementary file 4 — Supplementary Figure 3 [file 41419_2019_1664_MOESM4_ESM.tif]

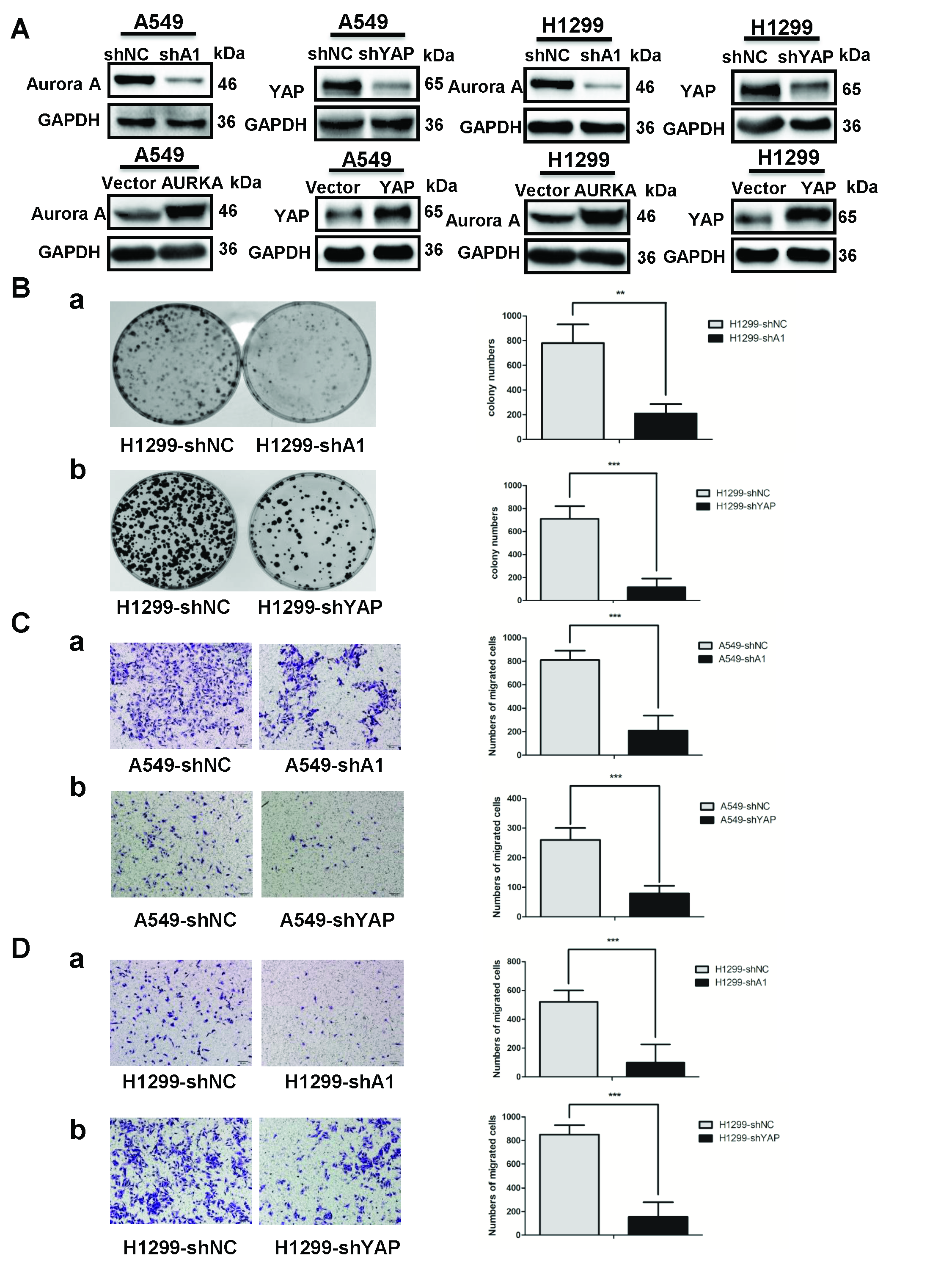

Supplement: Supplementary file 5 — Supplementary Figure 4 [file 41419_2019_1664_MOESM5_ESM.tif]

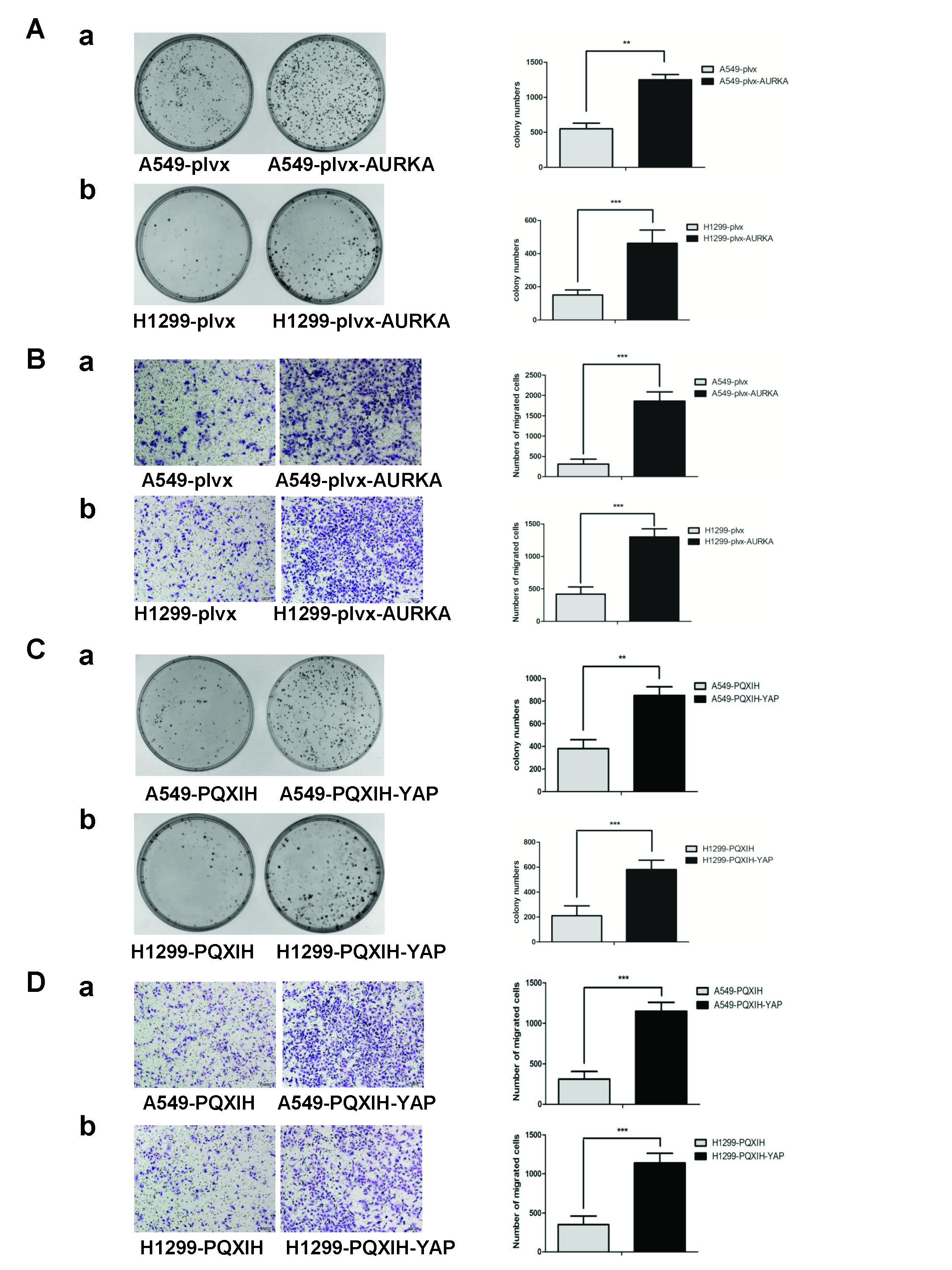

Supplement: Supplementary file 6 — Supplementary Figure 5 [file 41419_2019_1664_MOESM6_ESM.tif]
