# Supplementary material for: Activation of Aurora A kinase increases YAP stability via blockage of autophagy
Source: Cell Death Dis. 2019 Jun 3;10(6):432. doi: 10.1038/s41419-019-1664-4 (PMC6547697; doi:10.1038/s41419-019-1664-4)
Supplement: Supplementary file 7 — Supplementary Table 1 [file 41419_2019_1664_MOESM7_ESM.docx]

**Supplementary Table 1 Clinicopathologic characteristics in 43 patients with non-small cell lung cancer**

| Variable | Number (%) |
| --- | --- |
| Total | 43 (100) |
| Sex | |
| Male | 23 (53.5) |
| Female | 20 (46.5) |
| Age | |
| ≤69y | 22 (51.2) |
| >69y | 21 (48.8) |
| Smoking | |
| Non-smoker | 16 (37.2) |
| Ex-smoker | 13 (30.2) |
| Smoker | 14 (32.6) |
| Differentiation | |
| Well | 25 (58.1) |
| Moderate | 16 (37.2) |
| Poor | 2 (4.7) |
| Lymph node metastasis | |
| Absent | 38(88.4) |
| Present | 5 (11.6) |
| Blood vessel invasion | |
| Absent | 40 (93.0) |
| Present | 3 (7.0) |
| TNM Stage | |
| I | 38 (88.4) |
| II | 4 (9.3) |
| III | 1 (2.3) |
| AURKA expression | |
| High expression | 26 (60.5) |
| Low expression | 17 (39.5) |
| YAP expression | |
| High expression | 24 (55.8) |
| Low expression | 19（44.2） |
